# Fabrication of Site‐Specific 3D Structures via Macroscopic Supramolecular Assembly for Spatially Controlled Alignment of Multiple Cells

**DOI:** 10.1002/advs.202502425

**Published:** 2025-08-11

**Authors:** Yuchen Liu, Rui Ming, Qian Zhang, Yuguang Wang, Yijing Liu, Yuriy G. Galyametdinov, Andrey Knyazev, Feng Shi, Fang Liu, Mengjiao Cheng

**Affiliations:** ^1^ State Key Laboratory of Chemical Resource Engineering Beijing Laboratory of Biomedical Materials Beijing Advanced Innovation Centre for Soft Matter Science and Engineering Beijing University of Chemical Technology Beijing 100029 China; ^2^ Department of General Dentistry II National Center for Stomatology National Clinical Research Center for Oral Diseases National Engineering Research Center of Oral Biomaterials and Digital Medical Devices Peking University School and Hospital of Stomatology No.22, Zhongguancun South Avenue, Haidian Beijing 100081 China; ^3^ Department of Physical and Colloid Chemistry Kazan National Research Technological University 68 Karl Marx street Kazan 420015 Russia; ^4^ China‐Japan Friendship Hospital East Yinghuayuan Street 2, Chaoyang District Beijing 100029 China

**Keywords:** 3D ordered structure, host/guest interaction, macroscopic supramolecular assembly, modular assembly, selective cell adhesion

## Abstract

The self‐assembly of micrometer‐to‐millimeter components, referred to as “macroscopic supramolecular assembly (MSA),” offers an efficient approach for constructing cell‐scale 3D bioactive structures with flexible modular designs. Compared with available 3D bio‐printing or conventional modular assembly of cell‐material units, MSA is advantageous in decoupling material preparation and cell loading processes by directing cell adhesion after the preparation of 3D structures, which minimizes the trade‐off between cell viability and material selection. But the challenge lies in efficient self‐sorting of different cells and spatially controlled cell distribution. Hence, MSA is combined with the surface chemistry of orthogonally specific peptides to different cells and magnetic manipulation, and fabricated 3D bioactive structures that direct cell sorting. Microscale polydimethylsiloxane (PDMS) components are modified with 1) Arg‐Glu‐Asp‐Val and Val‐Ala‐Pro‐Gly peptides affinitive to endothelial cells (ECs) and smooth muscle cells (SMCs), respectively, and 2) host/guest molecules as “supramolecular glues” for precise structuring and interfacial bonding. Self‐sorting and spatially controlled adhesion of ECs and SMCs is achieved to mimic layered vascular structures. This “Lego‐like” strategy is free of compromising cell viability with structure design, thus contributing to spatially intricate and bioactive 3D architectures, and promoting the development of MSA from fundamental advances to applications.

## Graphical abstract

A modular macroscopic supramolecular assembly is developed to prepare 3D bioactive structures with self‐sorting of two primary vascular cells in post‐adhesion based on orthogonally affinitive peptides, thus minimizing the compromise in structuring and cell viability. Mild host/guest molecular recognition with controlled interfacial assembly kinetics acts as a flexible supramolecular glue for precise structuring aided by magnetic pick‐and‐place.

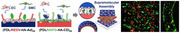

## 1 Introduction

3D bioactive constructs, comprising ordered biomaterials, site‐specific bioactive species, and precisely aligned multiple cell types, have gained significant attention in fields such as bioelectronics,^[^ ^1^, ^2^ ^]^ tissue engineering,^[^ ^3^, ^4^ ^]^ disease modeling, and drug screening.^[^ ^5^, ^6^, ^7^ ^]^ The spatial control over cell distribution in 3D constructs is crucial for creating precise living microenvironments that facilitate the study of cell‐material interactions.^[^ ^8^, ^9^ ^]^ Significant progress has been made in the fabrication of such bioactive 3D constructs, with increasing levels of spatial control.^[^ ^10^, ^11^ ^]^ Traditional methods, such as freeze‐drying or electrospinning biocompatible polymers (e.g., gelatin),^[^ ^12^ ^]^ result in randomly porous structures with minimal control over pore distribution or surface chemistry, leading to a non‐specific distribution of cells. With the advancement of additive manufacturing techniques like 3D bioprinting,^[^ ^13^ ^]^ cells can be precisely placed at designated positions, allowing for the alignment of different cell types on demand.^[^ ^6^ ^]^ Despite this, post‐crosslinking of precursor materials is required to stabilize the structure stepwise, limiting the choice of materials to those that are photo‐ or thermally curable.^[^ ^14^ ^]^ Additionally, printing methods involve trade‐offs between structural resolution and material selection, influenced by factors such as cell viability during printing, and the biocompatibility between cells and pre‐cured materials.^[^ ^15^ ^]^

To decouple the processes of structure formation and cell distribution, the modular assembly method introduces a “Lego‐like” fabrication approach.^[^ ^16^, ^17^ ^]^ This involves pre‐encapsulating cells in hydrogel blocks, which are then stacked to form 3D structures.^[^ ^18^, ^19^ ^]^ By loading different cell types into specific hydrogel blocks, on‐demand 3D alignment can be achieved, with cells remaining free from shear damage that could occur in extrusion‐based printing methods. However, trade‐offs still exist between cell viability and the crosslinkable properties of the encapsulating material.^[^ ^20^ ^]^ Ideally, instead of passively placing cells, active strategies should be developed to enable cells to automatically adhere to designated positions in prepared 3D structures with high selectivity. Such strategies would decouple the structure preparation and cell loading processes to expand the material versatility without compromising cell viability. However, the challenge lies in creating anisotropic surface chemistry within the 3D structures to tailor site‐specific affinity for different cell types.^[^ ^11^ ^]^ This requires both innovative 3D structuring principles and orthogonally selective surface chemistry.

Macroscopic supramolecular assembly (MSA) is an emerging 3D manufacturing approach that integrates cell‐scale modules into ordered structures through mild, non‐covalent supramolecular interactions^[^ ^21^, ^22^, ^23^, ^24^, ^25^, ^26^ ^]^ as opposed to traditional covalent post‐crosslinking. The primary steps in MSA include: 1) preparation of building blocks with precise control over size and shape, facilitated by MSA's compatibility with a wide range of processing techniques; 2) engineering of surface chemistry to introduce complementary supramolecular groups on the building blocks; and 3) interfacial assembly of the blocks into a designed geometry through both precise alignment and efficient supramolecular interactions. As a result, MSA inherits the advantages from the modular assembly strategy in flexible design and integration of diverse components, but with less dependence on the material types of the building blocks. Moreover, the assembly process can be enhanced by external alignment methods, such as capillary forces or magnetic fields,^[^ ^27^, ^28^, ^29^ ^]^ which aid in the precise positioning of building blocks ranging from micrometers to millimeters. The key to fabricating bioactive 3D constructs using MSA lies in the precise assembly of building blocks and the spatially selective post‐adhesion of cells.

In this study, we established a modular design and integration strategy by applying orthogonal peptide binding chemistry and combining MSA with magnetic pick‐and‐place techniques. This approach enables the creation of stacked, layered 3D bioactive constructs with spatially controlled distribution of multiple cell types. Biocompatible, microscale polydimethylsiloxane (PDMS) in long‐strip shapes were used as building blocks and embedded with Fe_3_O_4_ nanoparticles for MSA facilitated by magnetic pick‐and‐place. As a model system, we utilized endothelial cells (ECs) and smooth muscle cells (SMCs), which are the primary cell types in blood vessels and are arranged in a layered pattern. The surface chemistry of the PDMS building blocks was engineered with both orthogonally affinitive peptides and supramolecular groups to enable selective cell adhesion and modular assembly, respectively. Specifically, the polypeptides Arg‐Glu‐Asp‐Val (REDV) and Val‐Ala‐Pro‐Gly (VAPG) were used as affinitive species to ECs and SMCs, respectively;^[^ ^30^, ^31^ ^]^ β‐cyclodextrin (CD) and adamantane (Ad) groups served as a “supramolecular glue”^[^ ^32^ ^]^ to associate PDMS building blocks during MSA. Modular PDMS modified with different surface chemistry were aligned in different layers in the 3D construct, resulting in orthogonally selective cell adhesion. Namely ECs specifically adhered to the PDMS layer with REDV, while minimally adhering to the layer with VAPG, and vice versa for SMCs. This strategy avoids the shear forces or encapsulation processes that could reduce cell viability in printing techniques or previous modular methods, offering a straightforward, “Lego‐like” approach for 3D structuring with flexible cell‐free modular design. Owing to the large‐dimensional pre‐products, this method may create large‐scale 3D constructs with biological and chemical diversity, facilitating the generation of complex microenvironments for cell‐material studies.^[^ ^1^, ^5^, ^11^ ^]^

## 2 Results and Discussion

### 2.1 MSA Design to Obtain 3D Structures with Spatially Controlled Cell Distribution

**Figure 1:**
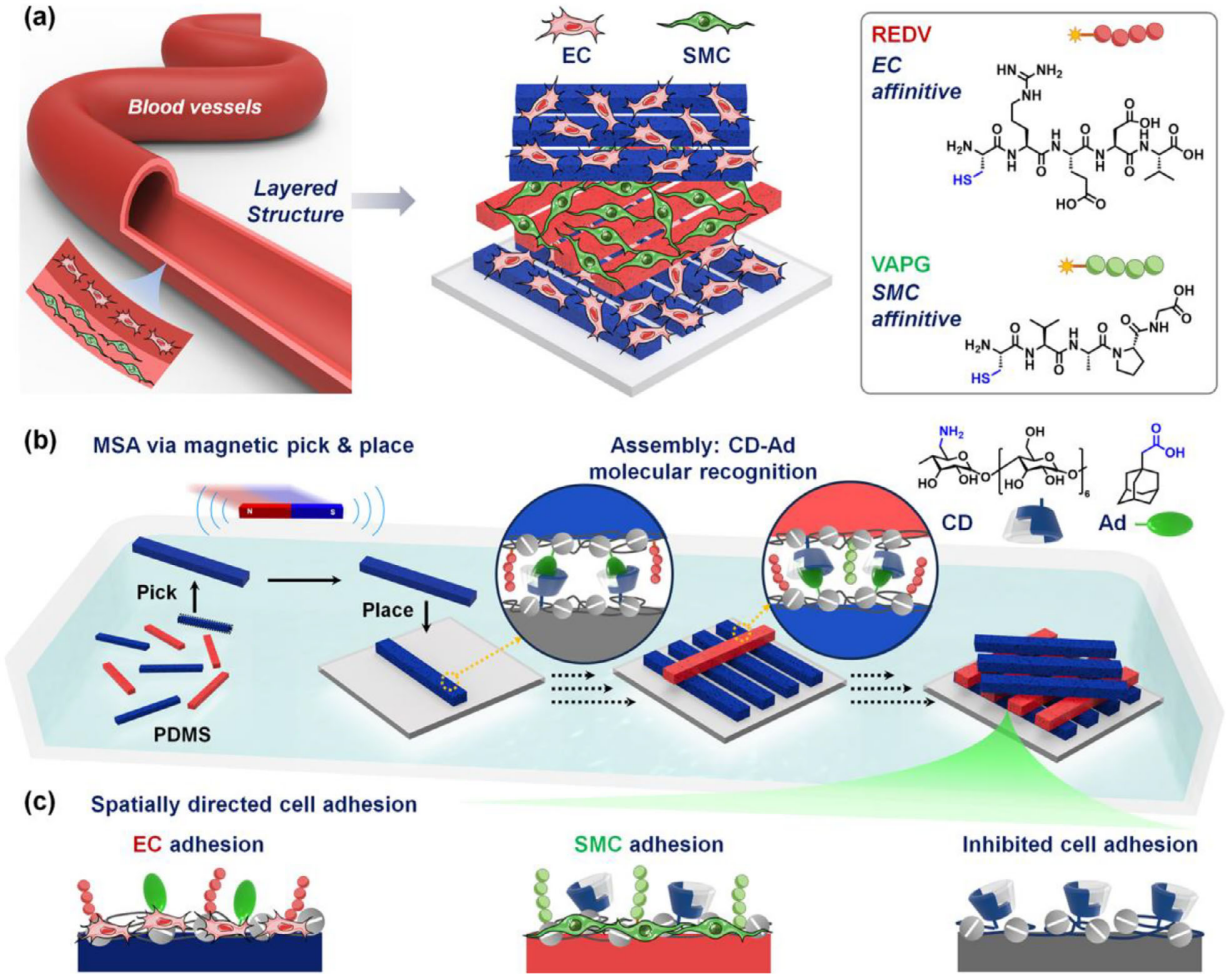
a) Schematic illustration of layered 3D bioactive constructs containing vascular cells of ECs and SMCs, which are affinitive to polypeptides of REDV and VAPG, respectively. b) Fabrication process of the 3D layered structure by combining MSA with magnetic pick‐and‐place of different PDMS building blocks which are non‐covalently assembled via molecular recognition between CD and Ad groups. c) Selective cell adhesion directed by the peptide surface chemistry of different PDMS.

Rather than pre‐encapsulating cells in specific materials and then stacking the cell‐material complex,^[^ ^20^ ^]^ MSA prefabricates 3D structures free of cells and then enables selective post‐adhesion of multiple cell types, thus avoiding the exposure of cells to chemical crosslinking during the material fabrication process. To demonstrate this proof‐of‐concept idea, we selected two primary vascular cell types, namely ECs and SMCs, to align them in a layered pattern (**Figure** **1**a). The key to achieving selective post‐adhesion lies in orthogonal peptide binding chemistry: ECs specifically bind to the REDV peptide,^[^ ^33^, ^34^ ^]^ while SMCs to the VAPG peptide,^[^ ^35^ ^]^ necessitating the modification of these peptides onto designated PDMS surfaces. Meanwhile, the fabrication of 3D layered structures requires both precise magnetic alignment of PDMS building blocks and a “supramolecular glue” to bond them, which involves incorporating magnetic‐responsive species and a host/guest molecular recognition pair of CD and Ad groups (Figure 1b). These multiple functionalities are realized by engineering PDMS building blocks with distinct surface chemistries: blue PDMS strips are modified with REDV and Ad groups, while red PDMS is modified with VAPG and CD groups (Figure 1c). The selectivity of cell adhesion is further enhanced by negatively charged hydrophilic surfaces that repel negatively charged cells; otherwise, positively charged surfaces would result in non‐selective cell adhesion because of their strong electrostatic attraction with negatively charged cells.^[^ ^36^ ^]^

### 2.2 Surface Engineering of PDMS Building Blocks with Multiple Functions

**Figure 2:**
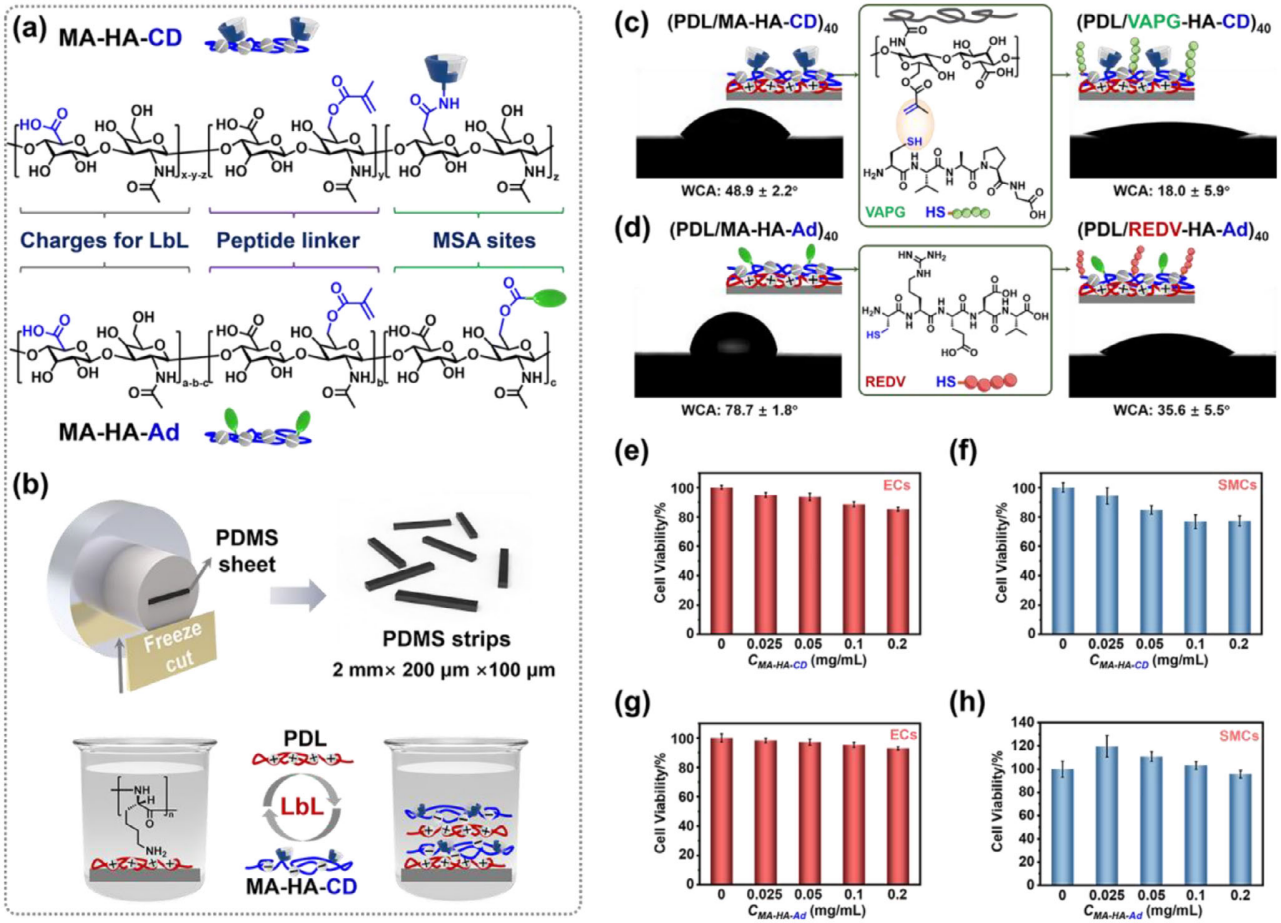
a) Polyelectrolytes to be modified onto PDMS: MA‐HA‐CD and MA‐HA‐Ad. b) Fabrication of PDMS strips as MSA building blocks and the LbL dipping process to modify oppositely charged polyelectrolytes of PDL and MA‐HA‐CD. Water contact angle (WCA) of PDMS surfaces after modified with LbL assembled polyelectrolyte multilayers of c) (PDL/MA‐HA‐CD)_40_ or d) (PDL/MA‐HA‐Ad)_40_, and after click with VAPG or REDV peptides. e–h) MTT results of ECs and SMCs in the presence of MA‐HA‐CD or MA‐HA‐Ad when varying their concentration.

The multiple functions of PDMS building blocks including selective cell adhesion and “supramolecular glue,” are realized by synthesizing a polyanion of hyaluronic acid (HA) derivative (**Figure** **2**a) consisting of multiple functional groups. First, the primary carboxylic acid groups on the HA backbone provide negative charges for the surface modification of PDMS via a layer‐by‐layer (LbL) technique^[^ ^37^ ^]^ by interacting with a polycation of poly‐D‐lysine (PDL) (Figure 2b). Second, partial substitution of HA with methacrylic anhydride (MA) groups as MA‐HA creates linking sites for peptides by thiol‐ene click reactions between methyl methacrylate groups on HA derivatives and thiol groups from peptides. Third, host or guest molecules, namely CD or Ad, have been grafted onto MA‐HA (Scheme S1, Supporting Information). The resulted MA‐HA‐CD (grafting ratio of CD: 7.5%; Figure S1a, Supporting Information) and MA‐HA‐Ad (grafting ratio of Ad: 13.6%; Figure S1b, Supporting Information) to be modified onto different PDMS, could act as MSA sites for further non‐covalent association between PDMS via host/guest molecular recognition, whose binding constant exceeds 10^4^ M^−1^, resulting in rapid wet adhesion following a multivalent mechanism in MSA.^[^ ^38^, ^39^ ^]^

The complete surface engineering of different PDMS building blocks was achieved by two procedures, namely the LbL self‐assembled procedure and the click chemistry to obtain modify peptide after LbL (Figure 1c,d). To introduce magnetic responsiveness, Fe_3_O_4_ nanoparticles were mixed in the pre‐polymer of PDMS, followed by freeze‐cutting the crosslinked PDMS as strips with a dimension of 2 mm × 200 µm × 100 µm (Figure S2, Supporting Information). Subsequently, the PDMS strips underwent an LbL dipping procedure to be modified with MA‐HA‐CD or MA‐HA‐Ad by alternate deposition with a polycation of PDL. The electrostatic attraction between positive charges on PDL and negatively charged carboxylic acid groups on HA, is the driving force for LbL. After 40 repeated dipping cycles, the resulted polyelectrolyte multilayers of (PDL/MA‐HA‐CD)_40_ and (PDL/MA‐HA‐Ad)_40_, exhibit a hydrophilic nature with a water contact angle (WCA) of 48.9° and 78.7° as shown in Figure 2c,d. Subsequently, the peptides of VAPG and REDV are introduced onto the PDMS surfaces via the click chemistry between MA and thiol groups, leading to reduced WCA to 18° and 35.6°, indicating that the hydrophobic MA groups have been replaced by the hydrophilic peptides.^[^ ^32^ ^]^ Besides, we have evaluated the toxicity of MA‐HA‐Ad and MA‐HA‐CD to ECs and SMCs with an MTT method. From Figure 2e–h, we observed that the cell viability remained over 90% below a concentration of 0.025 mg mL^−1^, which is a enough safe upper limit because the content of deposited polyelectrolytes in thin LbL multilayers (thickness: 20–26 nm; Figure S3, Supporting Information) is below the level of 0.001 mg mL^−1^.^[^ ^40^ ^]^ The film stability of the as‐prepared (PDL/VAPG‐HA‐CD)_40_ and (PDL/REDV‐HA‐Ad)_40_ multilayers on PDMS was evaluated by immersing in a solution of phosphate buffered saline for 48 h. The PDMS surfaces remain hydrophilic after the immersion (Figure S4, Supporting Information), indicating sufficient stability for further cell culture.

### 2.3 Fabrication of Precise 3D Structures via Controlled MSA

**Figure 3:**
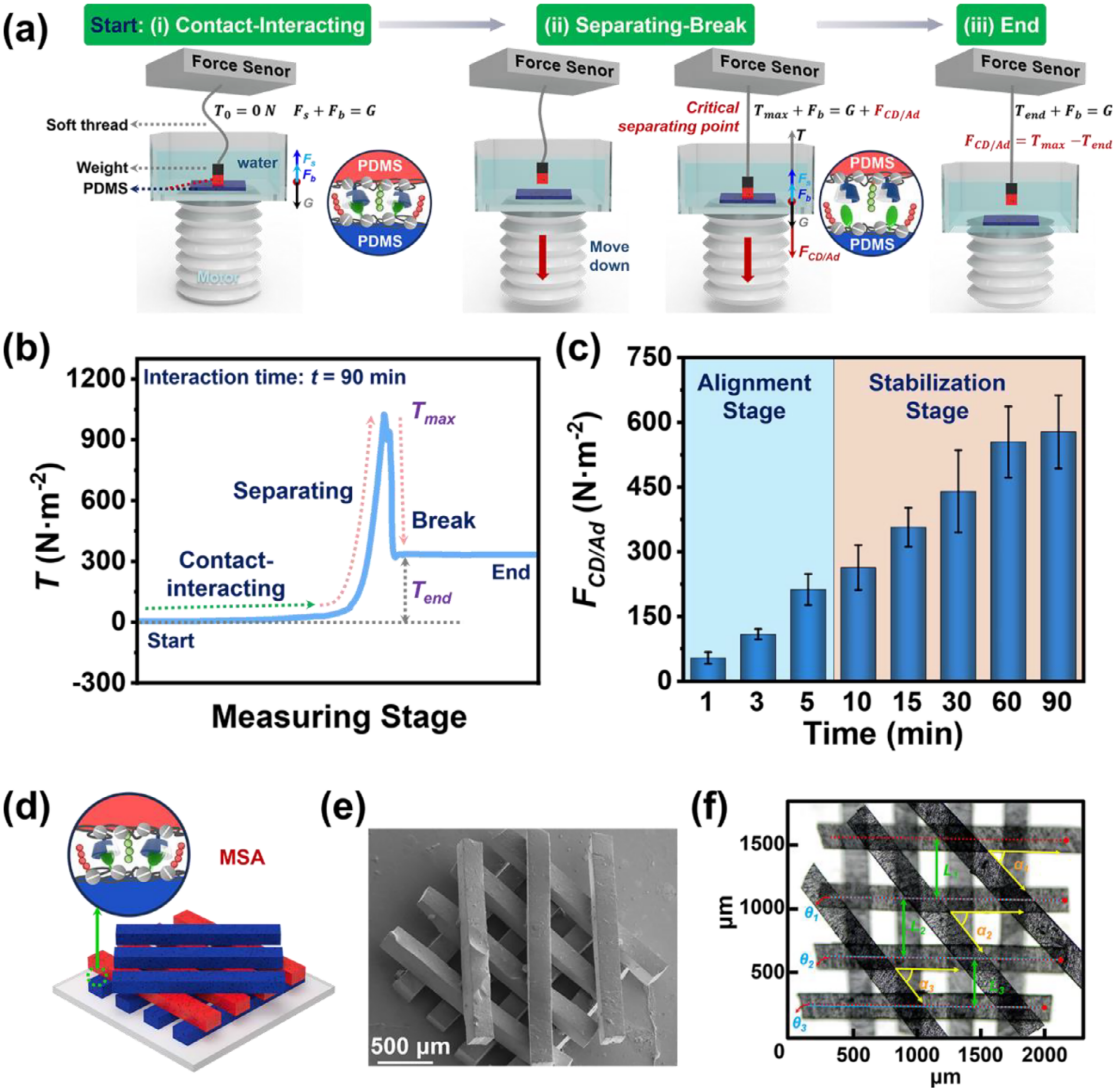
a) Illustration of in situ measurement of the interfacial CD/Ad molecular interaction between PDMS building blocks following a contact‐separating mode. b) A typical force curve after the PDMS has interacted for 90 min. c) Interfacial forces based on CD/Ad molecular interaction versus the interaction time. d) Illustration, e) SEM image, and f) optical microscopy image of a 3D layered structure assembled based on the CD/Ad molecular interactions between PDMS strips. The assembly precision is evaluated via parameters such as parallel degree, spacing, or twisting angle.

The fabrication of 3D stacked structures with good precision relies on both flexible adjustment of PDMS building blocks and stabilization of assembled structures, which are paradoxical to some degree. If the interfacial interactions between PDMS are too strong, fast stabilization of structures can be realized but delicate structural adjustment is not possible. However, if the interactions are weak, the stepwise construction via MSA combined with magnetic pick‐and‐place, will be infeasible because the unstable structure assembled in the last step might be damaged upon magnetic manipulation in the next step.^[^ ^21^ ^]^ The spatiotemporal control over the interfacial interactions in MSA is important in stepwise 3D structuring for the assembly method. Therefore, we studied the assembly kinetics of CD/Ad molecular recognition between two PDMS in a dynamic separating process with a force measurement apparatus as schematically illustrated in **Figure** **3**a. The upper force detector is sensitive to a weight change of microgram level and the bottom motor has a stepping precision of 0.01 mm s^−1^. Before the force measurement, we stacked a PDMS cube (red color shown in Figure 3a‐i) on a large PDMS sheet (blue color) to ensure a constant contacting area in repeated tests; the cube was connected to the upper force detector and the sheet was adhered to the bottom motor.

The whole measurement was conducted in water to match the MSA experimental condition. As the density of PDMS and water is close, we adhered a weight (a black cube) to the PDMS cube to avoid its buoyancy exceeding its gravity and hindering the interfacial interaction with the PDMS sheet. For simplicity, we considered the weight and the PDMS cube as an entity. After zeroing, the force changes in the thread could be in situ recorded as a pulling force (*T*) versus the motor position that indicates different measuring stages (Figure 3b). Taking the force curve after static interaction for 90 min as an example, the thread is loose without any tension (*T_0_* = 0 N) in the beginning. The forces exerted on the PDMS cube include gravity (*G*), buoyancy (*F_b_*), and supporting force (*F_s_*) from the motor. Then the thread gradually reaches a tensioned state where interface delamination occurs between the PDMS cube and sheet (Figure 3a‐ii). The resulted forces increase slowly and peak when the thread is fully tensioned (*T_max_*) to balance the downward interfacial molecular interaction and the gravity/buoyancy of PDMS. Finally, the contact between the PDMS cube and sheet breaks to result in their complete separation (Figure 3a‐iii). The residual force (*T_end_*) on the thread indicates the difference of gravity and buoyancy to be subtracted. As a result, the interfacial force contributed from CD/Ad molecular recognition could be calculated from the difference between *T_max_* and *T_end_*.

Following the above method, we have obtained the interaction kinetics of CD/Ad interactions between PDMS building blocks as summarized in Figure 3c. The interfacial binding force remains low (<200 N m^−2^) in the early stage below 10‐min stacking, thus allowing for timely disassembly to adjust the alignment of the PDMS strip if its precision is not satisfactory upon contacting. When the interaction time is increased to above 10 min, the assembled interface is stable enough with a force value above 300 N m^−2^ to resist disturbance from magnetic pick‐and‐place or external collision.^[^ ^41^ ^]^ After 90‐min stacking, the structure could be further stabilized with a stronger force levelling off at ≈550 N m^−2^, which is almost fives times of the control groups (Figure S5, Supporting Information) and comparable to the value (531.8 ± 100.9 N m^−2^) if the assembled building blocks are dried. The reason for the strong interfacial molecular recognition to associate large PDMS components is understood by a multivalent mechanism:^[^ ^42^ ^]^ numerous interfacial sites gradually achieve efficient multiple binding to improve the interfacial forces when the compliant surfaces and most motile molecules realize a self‐adaptive process to reach the interactive distance. This assembly kinetics depending on the contacting time between building blocks could address the paradox of dynamic structure adjustment (weak interfacial interactions are needed to dissociate imprecise structures) and its stabilization (strong interfacial binding is necessary) to achieve both precise and stable 3D stacked structures. If the binding force is too strong from the beginning, the structure adjustment would be difficult as the interacted interface is “locked” and “frozen” by strong interactions. Weak non‐covalent interactions allow for iterative adjustments or self‐correction to achieve precise structures.

In practice, we prepared two kinds of PDMS strips with complementary host/guest surface chemistry, namely surfaces with polyelectrolyte multilayers of either (PDL/REDV‐HA‐Ad)_40_ or (PDL/VAPG‐HA‐CD)_40_ containing peptides that are specifically affinitive to ECs and SMCs. To minimize cell adhesion on supporting substrates, we have modified quartz substrates with an LbL polyelectrolyte multilayer consisting of PDL and carboxylated chitosan (CCS) grafted with CD, noted as (PDL/CCS‐CD)_40_, which showed low cell adhesion owing to the right‐handed PDL and the highly hydrophilic CCS‐CD (Figure S6, Supporting Information).^[^ ^36^ ^]^ Following the procedure shown in Figure 1b, we picked the PDMS strip of (PDL/REDV‐HA‐Ad)_40_ on water and moved it to the (PDL/CCS‐CD)_40_ quartz with a permanent magnet; meanwhile, we gently removed water to result in the close contact between the PDMS strip and quartz assisted by magnet mooring. Precise positioning and refining of the PDMS strip on designated sites is realized by magnetic manipulation in ≈5–10 min, during which the CD/Ad interaction is weak for disassembly and re‐alignment of PDMS. Furthermore, the stabilization of the assembled PDMS is realized by longer interaction according to the assembly kinetics in Figure 3c. More PDMS strips could be assembled in a similarly stepwise manner into designated patterns, e. g., parallel strips as the first layer. The second layer was constructed based on the interfacial interactions between CD and Ad groups pre‐modified on the above two kinds of PDMS strips (Figure 3d). Finally, we obtained three‐layered stacked structures with parallel strips in the same layer but twisting for a certain angle between adjacent layers (e.g., 90° for the second layer and 45° for the third layer) (Figure 3e). Such a regularly porous structure may facilitate nutrient transport for cell growth.^[^ ^43^ ^]^

The assembly precision of the as‐prepared 3D structure is evaluated with parameters such as the parallel degree of PDMS strips in the same layer, spacing between adjacent PDMS, or twisting angle between layers by the ImageJ software. For the second layer of horizontally parallel PDMS, the parallel degree is measured as the deviation of the central axis (red dotted lines) of other strips relative to that of the first PDMS strip, and the averaged value is calculated to be *θ* = 0.2 ± 0.3°. The spacing between two adjacent strips (marked with red arrows) is controlled as *L* = 435.1 ± 48.3 µm, whose deviation is probably caused by the capillary attraction as the two PDMS approach.^[^ ^27^ ^]^ The twisting angle between the second and third layer is *α* = 49.6 ± 3.5°, exhibiting a high angle precision of below 5°. The combination of controlled interfacial interactions between PDMS and careful magnetic adjustment, contributes to on‐demand 3D alignment of each modular building block under mild and biocompatible aqueous solutions. The CD/Ad molecular recognition is favorable in the presence of water as the driving force for the molecular interaction is minimizing the high free energy of trapped water inside the hydrophobic CD cavity and outside the hydrophobic Ad molecules through the inclusion of Ad to CD.

Although stacking the PDMS strips one by one seems “low‐efficiency” compared with large quantity production, this modular approach is actually advantageous in addressing on‐demand fabrication requirements by considering both massive production and personalized demands. Most standard modules such as PDMS strips could be manufactured via conventional massive production techniques such as photolithography, moulding, which shows good fabrication efficiency. For further fabrication of unique 3D structures with personalized requirements such as tissue scaffolds, this approach provides flexible combination and alignment choices regarding module types, surface chemistry, surface biology etc. Thus, the method has combined the advantages of top‐down methods in massive production and those of bottom‐up methods in modular assembly. Possible future improvements to speed up the fabrication includes 1) applying an automatic magnetic manipulation system to realize both fast and parallel preparation, and 2) combining robotic vision intelligence with supramolecular assembly to achieve precise production independent on human labor.

### 2.4 Selective Adhesion of ECs and SMCs Realized by Orthogonal Peptide Chemistry

**Figure 4:**
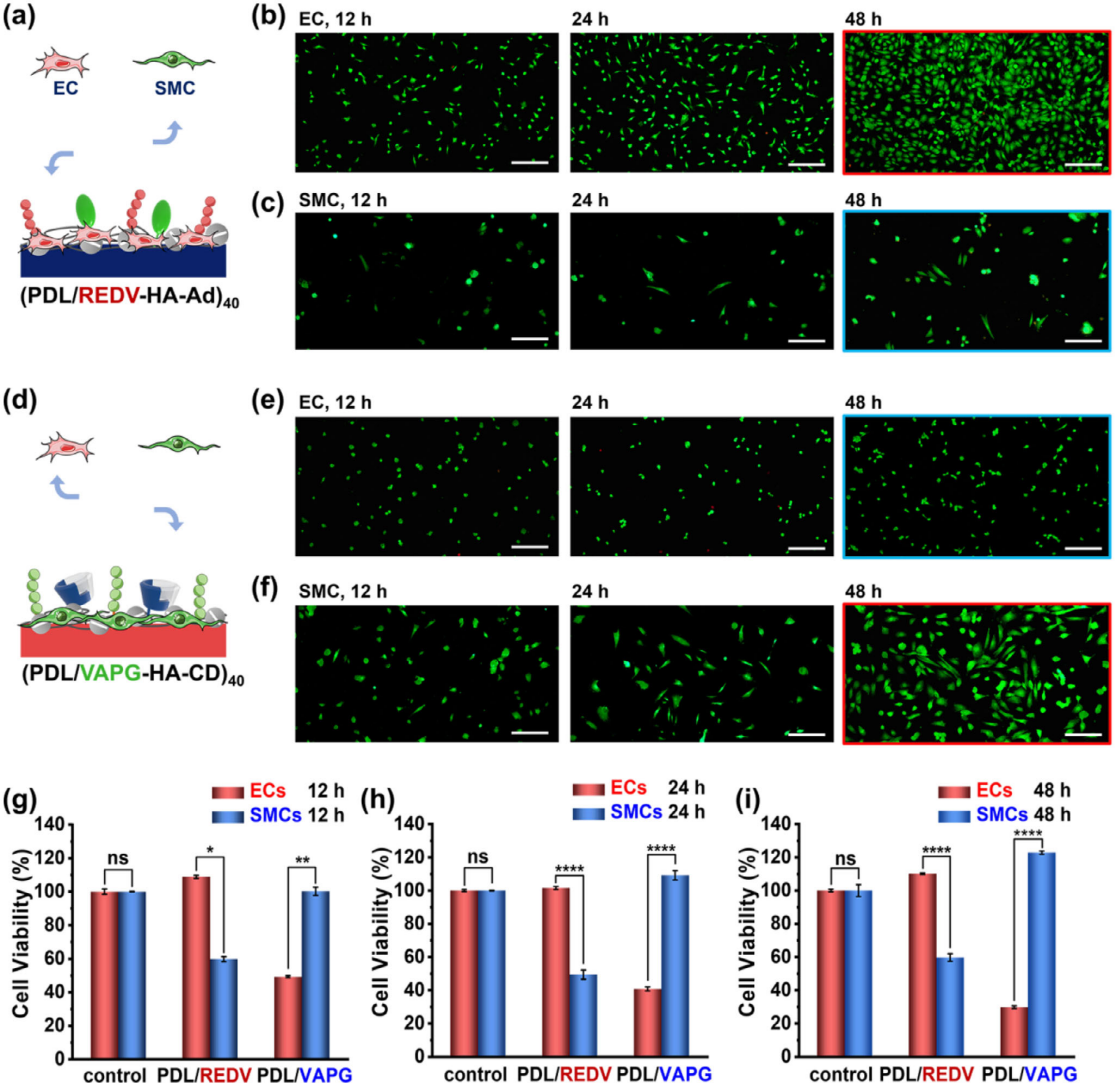
Schematic illustration and cell culture results of ECs and SMCs displaying selective adhesion on surfaces with different surface chemistry: a–c) (PDL/REDV‐HA‐Ad)_40_ and d–f) (PDL/VAPG‐HA‐CD)_40_. Scale bars: 200 µm. Cell viability evaluated with CCK‐8 assays after culture of ECs and SMCs for g) 12, h) 24, and i) 48 h on varied surface chemistry: substrates modified with (PDL/REDV‐HA‐Ad)_40_, (PDL/VAPG‐HA‐CD)_40_ multilayers noted as PDL/REDV and PDL/VAPG in short; cleaned quartz substrates are used as control. Data are shown as mean ± S.D. Statistical analysis: one‐way ANOVA with Tukey's test (^*^ *p* <0.05, ^**^ *p* <0.01, ^***^ *p* <0.001, ^****^ *p* <0.0001; ns, not significant).

After achieving the fabrication of 3D structures with control over spatially diverse surface chemistry, it remains to prove the hypothesis of selective cell adhesion on the designated surfaces or structures. Therefore, we tried to clarify the effects of orthogonal peptide surface chemistry on selective cell adhesion by comparing the growth and proliferation of of both ECs and SMCs on the surfaces of (PDL/REDV‐HA‐Ad)_40_ and (PDL/VAPG‐HA‐CD)_40_, respectively. For each kind of surface chemistry, we seeded and cultured ECs or SMCs for 12, 24, and 48 h under similar conditions, followed by live/dead staining with the assay of Calcein‐AM and propidium iodine (PI) indicating live cells in green colors and dead one in red colors. The results of cell adhesion on (PDL/REDV‐HA‐Ad)_40_ surfaces are summarized in **Figure** **4**a–c: the live ECs are relatively smaller oval‐shaped while the live SMCs have a larger size and an elongated shape; at each culture time, the cell density of ECs is always much larger than that of SMCs, indicating a better affinity of this REDV surface toward ECs; with increasing culture time, the density of ECs exhibit a growing trend to cover the surfaces thoroughly (Figure 4b) while the SMCs density remains low (Figure 4c). Hence, the applied surface chemistry of (PDL/REDV‐HA‐Ad)_40_ displays the 2D selectivity of ECs over SMCs when cultured individually. Besides selectivity, the biocompatibility of the material system is well maintained as the slightly adhered SMCs also exhibit a healthy state.

Similarly, we have changed the surface chemistry as (PDL/VAPG‐HA‐CD)_40_ and evaluated the adhesion behaviors of ECs and SMCs (Figure 4d). The results are opposite: little proliferation of ECs was found with increasing culture time (Figure 4e) probably due to the absence of affinitive species of REDV and the negatively charged multilayers repelling the cell adhesion to some degree. For SMCs, this VAPG surface is affinitive to result in high‐density cell coverage and observable proliferation (Figure 4f). The above observable selective cell adhesion matches well with the quantified results of the CCK‐8 assay that indicates the cell viability with the above surface chemistry under similar culture conditions. The control groups contain the culture medium, CCK‐8, and blank quartz substrates while the experimental groups contain substrates with REDV or VAPG surface chemistry. After staining, the absorbance of each control group was calculated as the base to compare the relative viability of experimental groups. The middle columns in Figure 4g–i representing the surfaces containing REDV peptides, show more than 50% higher ECs than SMCs, indicating the selective affinity to ECs; on the contrary, the right columns have demonstrated that the VAPG surfaces specifically bind SMCs with over 70% higher viability (48 h) over ECs. The above difference in cell density is significance according to the statistic analysis. The 2D orthogonal selectivity of both cells toward corresponding specific surface chemistry, provides the potential to realize self‐sorting adhesion when co‐culture in 3D structures.

The above selective adhesion results are mainly attributed to the minimization of non‐specific adhesion via two designs: 1) the co‐existence of both cell‐affinitive and cell‐repulsive species on the multilayers of PDMS; 2) applying the chiral selectivity of PDL according to our previous work.^[^ ^36^ ^]^ In the first design, the surface chemistry of (PDL/VAPG‐HA‐CD)_40_ and (PDL/REDV‐HA‐Ad)_40_ multilayers on PDMS, is “contradictory” with both cell‐affinitive species (e.g., VAPG specifically affinitive to SMCs or REDV to ECs) and cell‐repulsive species of HA‐CD or HA‐Ad as the outmost groups, whose negatively charged nature is similar to that of cell surfaces and thus making the cell adhesion not favorable. With this design, non‐specific cell adhesion could be minimized because no affinitive species are present on the PDMS for non‐specific cells. Taking the (PDL/VAPG‐HA‐CD)_40_ designed to specifically absorb SMCs as an example, the presence of VAPG facilitates the adsorption of SMCs while HA‐CD hinders the adhesion, indicating a chance that affinitive VAPG wins to result in the adhesion of SMCs; however, this surface chemistry has no affinitive species for ECs and thus the cell‐repulsive HA‐CD could reduce the non‐specific adhesion. In the second design, we found that levorotatory polylysine, namely poly‐L‐lysine (PLL), was more affinitive to cell adhesion than dextrorotatory PDL when both enantiomeric forms were present. Hence here we used the less cell‐affinitive PDL rather than PLL as the polycation for the multilayer construction, to minimize non‐specific cell adhesion.

### 2.5 3D Selectivity of ECs and SMCs in Co‐Culture

**Figure 5:**
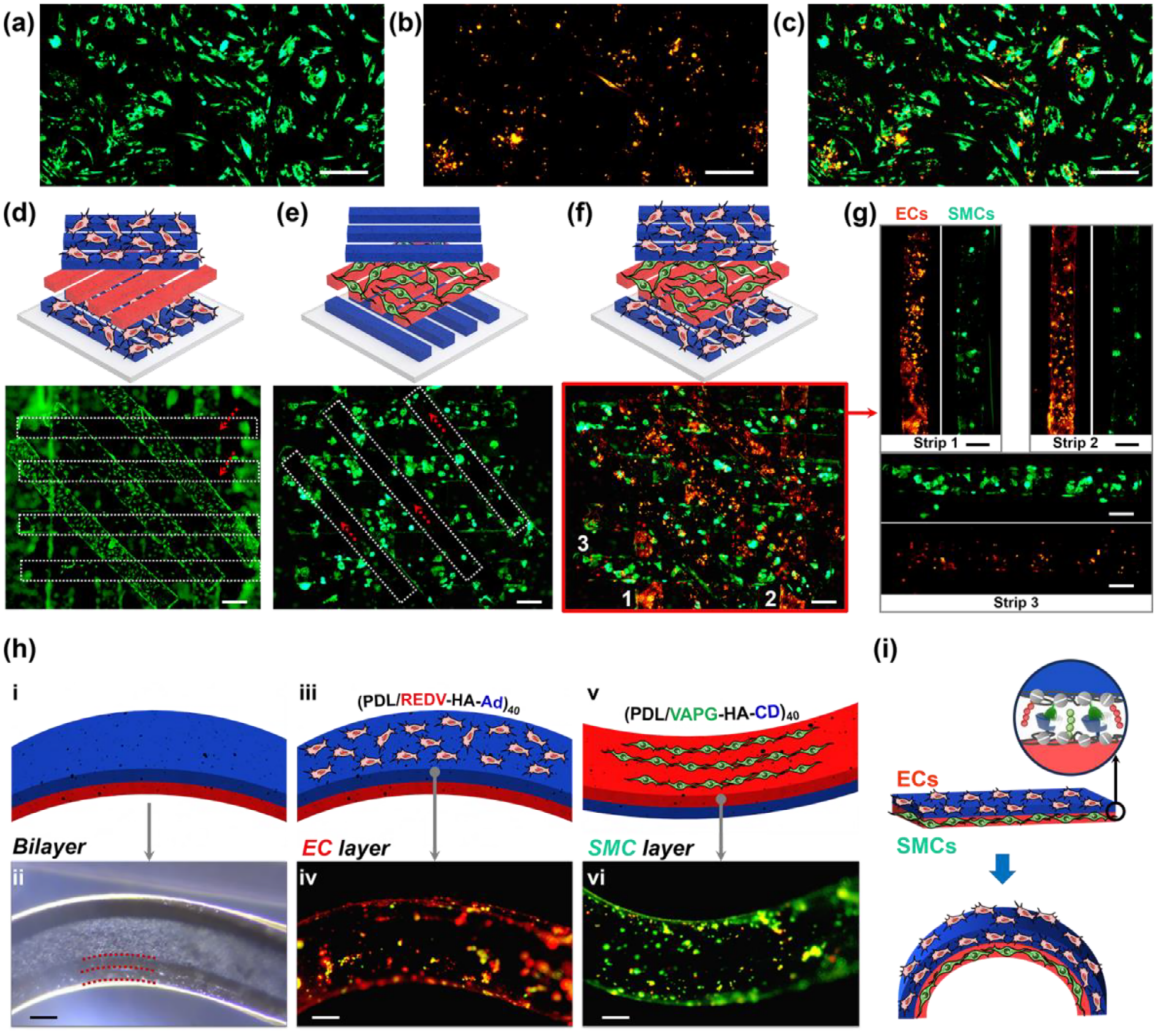
Fluorescent images after co‐culture of ECs and SMCs on 2D substrates with the surface chemistry of (PDL/VAPG‐HA‐CD)_40_ multilayers: a) DiO‐labeled SMCs, b) Dil‐labeled ECs, and c) merged. Fluorescent images showing 3D selectivity of ECs and SMCs in 3D PDMS structures with the top and bottom layer modified as (PDL/REDV‐HA‐Ad)_40_, and the middle layer as (PDL/VAPG‐HA‐CD)_40_: culture of d) only ECs or e) only SMCs (stained with Calcein‐AM/PI), and f) co‐culture of Dil‐labeled ECs and DiO‐labeled SMCs; g) single strips marked as 1–3 in (f) before merged. h,i) Co‐culture of labeled cells on a tubular bilayer PDMS structure with different surface chemistry: i‐ii) shows the schematic illustration and optical microscope image, iii‐iv) shows the upper layer (with REDV) and its fluorescent image of ECs, and v‐vi displays the bottom layer (with VAPG) and its fluorescent image of SMCs. Scale bars: 200 µm.

The spatially controlled alignment of multiple cells in 3D structures require two primary factors: 1) selectivity in co‐culture and 2) 3D selectivity to overcome the gravity‐directed adhesion for conventional cell seeding methods. For the culture of mixed cells, the surface chemistry should satisfy both cell compatibility and meanwhile selectivity in adhesion and growth. To clarify the first point of co‐culture selectivity, we applied the Dil/DiO fluorescent dyes that are primarily used for labeling cell membranes to distinguish ECs (exhibiting an orange fluorescent color after labeling) and SMCs (green), respectively. The two kinds of labeled cells were mixed and seeded together onto the surfaces modified with (PDL/VAPG‐HA‐CD)_40_ or (PDL/REDV‐HA‐Ad)_40_ multilayers. From the results in **Figures** **5**a–c and S7 (Supporting Information), we could observe that both cells stay alive in the applied surface chemistry, indicating the biocompatibility of all chemicals; meanwhile, the adhesion selectivity is significant with SMCs having a much higher density from the comparison of single fluorescence images of the two cells (Figure 5a,b) or their merged image (Figure 5c). Statistically significant differences were analyzed using one‐way analysis of variance (ANOVA) as summarized in Figure S8a (Supporting Information). This phenomenon has confirmed the feasibility of co‐culture selectivity of ECs and SMCs directed by the orthogonally effective VAPG and REDV peptides.

Considering that normal gravity‐based seeding may also direct cell adhesion, we have fabricated 3D layered structures of stacked PDMS strips with varied surface chemistry in the vertical direction, and studied the 3D affinity of ECs or SMCs. The 3D PDMS structures have the first and third layer modified with (PDL/REDV‐HA‐Ad)_40_ multilayers and the middle layer with (PDL/VAPG‐HA‐CD)_40_ multilayers, which are designated to bind ECs and SMCs accordingly. Even though the vertical distance of the three‐layer structure is almost 300 µm (more than 30 times of the cell size), spatially directed adhesion in this vertical distance could still be observed in Figure 5d: the density of ECs on the bottom and top layer (45 degree to the bottom layer) is higher than the second layer of PDMS strips, whose contours are marked with white dotted frames and the surfaces are marked with red arrows indicating little adhesion of ECs. On the contrary, when applying the same 3D structure for the culture of SMCs, the results are opposite: the middle layer of horizontally aligned PDMS strips containing VAPG have more SMCs while the top layer with REDV is almost empty with little cell adhesion; the bottom layer also displays low‐density cell adhesion probably because of a slight gravity‐based seeding effect and the biocompatibility of the applied multilayers. Hence, the spatial adhesion selectivity of either ECs or SMCs in 3D structures with a high vertical difference of more than 300 µm, functions well with the REDV and VAPG peptide surface chemistry in the presence of the gravity effect.

Further 3D selectivity in co‐culture has been demonstrated by Dil‐labeled ECs and DiO‐labeled SMCs in a 3D PDMS structure. As displayed in the merged fluorescent image in Figure 5f, the layered structure is alternately colored with the bottom and top layer in an orange red color and the middle layer in a green color, indicating the spatially selective distribution of ECs and SMCs toward the REDV and VAPG specific layers. The spatial selectivity is relative based on the comparison of cell density, e.g., the bottom and top layer have more ECs than SMCs. For better observation, we have isolated some strips from the merged 3D structures in Figure 5f to show each cell density on single strips in Figure 5g: Strip 1 and 2 from the bottom layer that is affinitive to ECs, and Strip 3 from the middle layer that is affinitive to SMCs. By comparing the fluorescence strength and distribution indicating the presence of ECs (orange red color) or SMCs (green), we found stronger ECs fluorescence than SMCs on both Strip 1 & 2, but lower signal on Strip 3, indicating the 3D selectivity of both cells under co‐culture conditions. Statistically significant differences were analyzed in Figure S8b (Supporting Information).

To mimic the vascular structure consisting of ECs and SMCs distributed in a layered manner, we have prepared a bilayer structure by attaching two PDMS sheets with CD/Ad supramolecular recognition, and conducted co‐culture of both cells (Figure 5h,i). The top layer was modified with EC‐affinitive (PDL/REDV‐HA‐Ad)_40_ and the bottom layer with SMC‐affinitive (PDL/VAPG‐HA‐CD)_40_. The two DiO/Dil‐labeled cell lines were mixed before seeding. The bilayer structures were flipped after seeding the cell mixture for 24 h to ensure both sides were seeded with cells. After gentle bending into a tubular structure, we observe the selective adhesion of ECs on the top layer and SMCs on the bottom layer, according to the comparison of cell fluorescence between single fluorescent images in Figure 5h (i–iii) and (iv–vi) before and after merge. Taken together, spatially controlled cell adhesion onto designated positions in 3D structures is feasible by post‐adhesion of co‐cultured cells, which thus could decouple the structure preparation and cell seeding processes for flexible modular design without scarifying cell viability in most modular pre‐encapsulation^[^ ^44^, ^45^ ^]^ or 3D bio‐printing strategies.^[^ ^15^ ^]^ Moreover, because each modular components are flexible for personalized design, other advanced factors that influence cell fates could be introduced to such 3D structures for understanding complex cellular mechanosensing mechanism such as creating surface micro‐grooves to explain cell polarization as demonstrated by Xu et al., regarding space constraint and adhesion induction.^[^ ^46^, ^47^ ^]^ The material choice is not limited to PDMS, which is applied as a model system. Other biocompatible materials such as hydrogel, poly(ε‐caprolactone), polylactic acid etc., are also applicable providing that the multilayers could be deposited onto surfaces. Owing to the flexibility in magnetic alignment and adjustment of interfacial interactions, the pore size could be tailored on demand, which is important for effective nutrients and waste exchange in culturing a large quantity of cells.

## 3 Conclusion

We have demonstrated a modular assembly strategy to prepare 3D bioactive structures with spatially controlled alignment of multiple cells by combining macroscopic supramolecular assembly with the surface chemistry of orthogonally specific peptides. PDMS strips were used as building blocks and engineered with multifunctional surface chemistry, namely (PDL/REDV‐HA‐Ad)_40_ and (PDL/VAPG‐HA‐CD)_40_ LbL assembled multilayers, for purposes of cell selectivity to ECs and SMCs, surface modification, and interfacial connection of PDMS, biocompatibility. Anisotropic 3D ordered structures with good spatial control was fabricated on demand following the “Lego‐like” MSA method together with magnetic pick‐and‐place. The interfacial host/guest molecular recognition acts as the “supramolecular glue” to bond different PDMS building blocks. The interfacial interactive force between PDMS with a dynamic interactive kinetics was in situ measured to offer the feasibility of spatiotemporal control over precise structuring mildly. The orthogonal 2D/3D cell selectivity in co‐culture has been demonstrated: ECs specifically adhere to the affinitive REDV PDMS layer but minimally to the non‐affinitive VAPG layer while SMCs show the opposite behavior.

This “Lego‐like” strategy is free of compromising cell viability with structure design, and provides abundant selection of modular surface chemistry, thus contributing to diverse interior/surface chemistry of 3D structures for potential uses in cell‐material interaction study for biosensing or implanting,^[^ ^48^, ^49^ ^]^ biohybrid actuators,^[^ ^50^ ^]^ tissue engineering such as skins or bones,^[^ ^51^, ^52^ ^]^ organ chips^[^ ^1^, ^3^ ^]^ etc. Especially, MSA provides solutions to tissue formation with advantages in being mild, biocompatible, flexible with modular design, compatible with massive production techniques, rich with chemically specific interactions for selective cell adhesion, and less dependent on material choices. Moreover, the demonstration of fabricating 3D bioactive structures has promoted the application of the emerging new research field of MSA by taking its advantages in mild and abundant modular design.^[^ ^21^, ^22^, ^23^, ^24^, ^25^ ^]^

## 4 Experimental Section

### Preparation of PDMS Strips and the Surface Modification

PDMS prepolymer solution (10 g), curing agent (1 g), and Fe_3_O_4_ magnetic nanoparticles (100 mg) were mixed mechanically for 15 min, degassed, and sandwiched between two glass slides with a spacing of ≈200 µm. After curing at 65 °C for 4 h, the cured PDMS was manually cut into a size of 2 mm × 2 mm × 200 µm, followed by slicing with a freezing microtome to the final dimension of 2 mm × 200 µm × 100 µm. The cleaned PDMS strips was treated with plasma as hydrophilic and then immersed in a PDL solution (aq, 0.5 mg mL^−1^) for 12 h. The subsequent LbL assembly of PDMS was conducted by alternate immersion in PDL (aq, 0.5 mg mL^−1^) and MA‐HA‐CD (aq, 1 mg mL^−1^) solutions for 5 min each, between which PDMS were cleaned with deionized water. The LbL procedure were repeated for 40 cycles to result in (PDL/MA‐HA‐Ad)_40_ or (PDL/MA‐HA‐CD)_40_ multilayers. Then the PDMS strips was immersed in a mixed solution of a peptide solution of REDV or VAPG (aq, 1 mg mL^−1^), a reducing agent of tris(2‐carboxyethyl) phosphine (150 µL), and a photo initiator of Ig 2959 (30 µL), followed by exposing to UV light for 10 min. The resulted surfaces were washed with deionized water and dried with N_2_ to obtain (PDL/REDV‐HA‐Ad)_40_ or (PDL/VAPG‐HA‐CD)_40_ multilayers.

### Fabrication of 3D Structures by MSA and Magnetic Pick‐and‐Place

A quartz substrate modified with the (PDL/CCS‐CD)_40_ multilayer was immersed in water, and PDMS strips modified with either (PDL/REDV‐HA‐Ad)_40_ or (PDL/VAPG‐HA‐CD)_40_ multilayers were distributed on water. Then a permanent magnet was applied to pick a (PDL/REDV‐HA‐Ad)_40_ PDMS to the designated position on the quartz, followed by removing excessive water with a filter paper to make the PDMS contact the substrate to trigger the interfacial CD/Ad interactions. If the positioning is not precise, water was re‐added and a magnet was applied to adjust. If precise, the structure was kept dry for 10 min after draining water. After stabilization without response to magnet movement, a new PDMS will be assembled following the above procedure. It took ≈2–3 h to complete a 3D structure with 11 strips, and assembling each strip took ≈15 min. Most time was spent in repeated adjusting the strip position to make the structure precise and waiting for the drying of apparent water to stabilize the structures.

### Cell Culture Conditions

ECs were cultured in DMEM supplemented with 10% FBS, 1% endothelial cell growth factor, and 1% P/S while SMCs in DMEM with 20% FBS and 1% P/S. Both cells were cultured at 37 °C with 5% CO_2_. Cell viability in the presence of polyelectrolytes was evaluated via an MTT method: ECs/SMCs (2.5 × 10^4^ cells well^−1^ in 96‐well plates) were treated with polyelectrolytes (MA‐HA‐Ad or MA‐HA‐CD; 0.025–0.2 mg mL^−1^) for 24 h. Then, MTT solution (5 mg mL^−1^ in PBS) was added, incubated for 4 h, and absorbance measured at 490 nm. 2D Selective Cell adhesion on varied surface chemistry was compared with the CCK‐8 assay: ECs (5 × 10^4^ cells well^−1^ in 24‐well plates) were seeded on polyelectrolyte‐modified quartz in 24‐well plates. After 12/24/48 h, CCK‐8 reagent was added, incubated for 2 h, and absorbance read at 450 nm. *Co‐culture of ECs and SMCs*: SMCs were pre‐stained with DiO (25 µm, 25 min) ECs with Dil (3 µm, 5 min); for staining the cell density is ≈10^6^ cells mL^−1^. The stained cells (ECs, SMCs, or 2:1 ECs/SMCs mix) were seeded on 2D substrates or 3D PDMS structures (ECs: 1 × 10^6^ cells well^−1^ and SMCs: 5 × 10^5^ cells well^−1^ in 12‐well plates) for 48 h. For bilayer PDMS structures: Mixed cells (ECs: 2 × 10^5^ cells well^−1^ and SMCs: 1 × 10^5^ cells well^−1^ in 48‐well plates) were seeded on one side (24 h), flipped, and reseeded on the opposite side (48 h).

## Conflict of Interest

The authors declare no conflict of interest.

## Supporting information



Supporting Information

## Data Availability

The data that support the findings of this study are available from the corresponding author upon reasonable request.
