# Supplementary material for: Fabrication of Site‐Specific 3D Structures via Macroscopic Supramolecular Assembly for Spatially Controlled Alignment of Multiple Cells
Source: Adv Sci (Weinh). 2025 Aug 11;12(33):e02425. doi: 10.1002/advs.202502425 (PMC12412592; doi:10.1002/advs.202502425)
Supplement: Supplementary file 1 — Supporting Information [file ADVS-12-e02425-s001.docx]

Supporting Information

**Fabrication of Site-Specific 3D Structures via Macroscopic Supramolecular Assembly for** **Spatially Controlled Alignment of Multiple Cells**

Yuchen Liu, Rui Ming, Qian Zhang, Yuguang Wang, Yijing Liu, Yuriy G. Galyametdinov, Andrey Knyazev, Feng Shi, Fang Liu*, and Mengjiao Cheng*

**Table of Content:**

[1. Materials and Instrument 3](#_Toc196202785)

[2. Synthesis of MA-HA-CD and MA-HA-Ad 4](#_Toc196202786)

[3. Fabrication of PDMS Strips 6](#_Toc196202787)

[4. Thickness of Polyelectrolyte Multilayers 6](#_Toc196202788)

[5. Film Stability of (PDL/VAPG-HA-CD)_40_ and (PDL/REDV-HA-Ad)_40_ Multilayers 7](#_Toc196202789)

[6. Interfacial Interactive Forces between Building Blocks of Varied Surface Chemistry. 7](#_Toc196202790)

[7. Cell Viability of CCS-CD and Cell Adhesion on (PDL/CCS-CD)_40_ Multilayers. 8](#_Toc196202791)

[8. Co-Culture of ECs and SMCs on (PDL/REDV-HA-Ad)_40_ Multilayers. 8](#_Toc196202792)

[9. Statistical Analysis of Significant Cell Adhesion Behaviors. 8](#_Toc196202793)

[10. Cell Distribution in the Bilayer Structure. 9](#_Toc196202794)

# 1. Materials and Instrument

The following chemicals were used as purchased:

Hyaluronic acid (HA), methacrylic anhydride (MA), ion exchange resin (Dowex 50W X8), tetrabutylammonium hydroxide (TBA-OH), di-*tert*-butyl dicarbonate (BOC_2_O), 3,3′-dioctadecyloxacarbocyanine perchlorate (DiO) and 1,1′-dioctadecyl-3,3,3′,3′-tetramethylindocarbocyanine perchlorate (Dil) from Sigma-Aldrich; 6-amino-β-cyclodextrin (CD) from Shandong Binzhou Zhiyuan Biotechnology; 1-adamantaneacetic acid (Ad), tris(2-carboxyethyl) phosphine (TCEP), photoinitiator 2959 (Ig 2959), *N*-(3-(dimethylamino)propyl)-*N′*-ethylcarbodiimide hydrchloride (EDC), and *N*-hydroxysuccinimide (NHS) from TCI; 4-dimethylaminopyridine (DMAP) were purchased from Alfa Aesar; carboxylated chitosan (CCS) and propidium iodine (PI) from Aladdin; PDMS (Sylgand 184) from Dow Corning; Fe_3_O_4_ magnetic nanoparticles from Beijing DK Nanotechnology; poly-D-lysine (PDL), Calcein-AM, and dialysis bag (3,500 Da molecular weight cutoff) Shanghai Yuanye Biotechnology; CREDV (Cys-Arg-Glu-Asp-Val) and CVAPG (Cys-Val-Ala-Pro-Gly) peptides from Bankpeptide Biological Technology; SMCs (rabbit aortic smooth muscle cells) from the Cell Resource Center, Peking Union Medical College; ECs (human umbilical vein endothelial cells) and ECM medium MeisenCTCC; DMEM medium and penicillin/streptomycin (P/S) from Gibco; PBS, trypsin, and MTT from Solarbio Life Sciences; fetal bovine serum (FBS) from Hyclone; Cell Counting Kit-8 (CCK-8) from Dojindo; cell culture consumables such as cell culture flasks (T-25 polystyrene flasks), culture microplates, and pipettes from Corning Incorporated.

^1^H-NMR spectra were measured with an NMR spectrometer (Bruker AV400) at 400 MHz at room temperature. PDMS building blocks were prepared by a freezing microtome (CM1950, Leica). MTT and CCK-8 results were obtained with a microplate reader (Multiskan FC, Thermo Fisher Scientific) at 490 nm and 450 nm, respectively. The interfacial force was measured with a dynamic contact angle tensiometer (DCAT21, Dataphysics). The thickness of polyelectrolyte multilayers was measured by atomic force microscopy (AFM) (Dimension 3100, Bruker). Water contact angle was measured with an optical contact angle measuring and contour analysis systems (OCA20, Dataphysics). Fluorescent images were obtained with a fluorescence microscopy (BX53, Olympus). Confocal laser scanning microscope (CLSM) (TCS SP8 X, Leica Microsystems) was used to show the cell distribution in the bilayer structure (green DiO for SMC cells, Ex/Em: 484/501 nm; orange Dil for EC cells, Ex/Em: 549/565 nm). The permanent magnet for magnetic pick-and-place has a field strength of about 1~2 mT.

# 2. Synthesis of MA-HA-CD and MA-HA-Ad


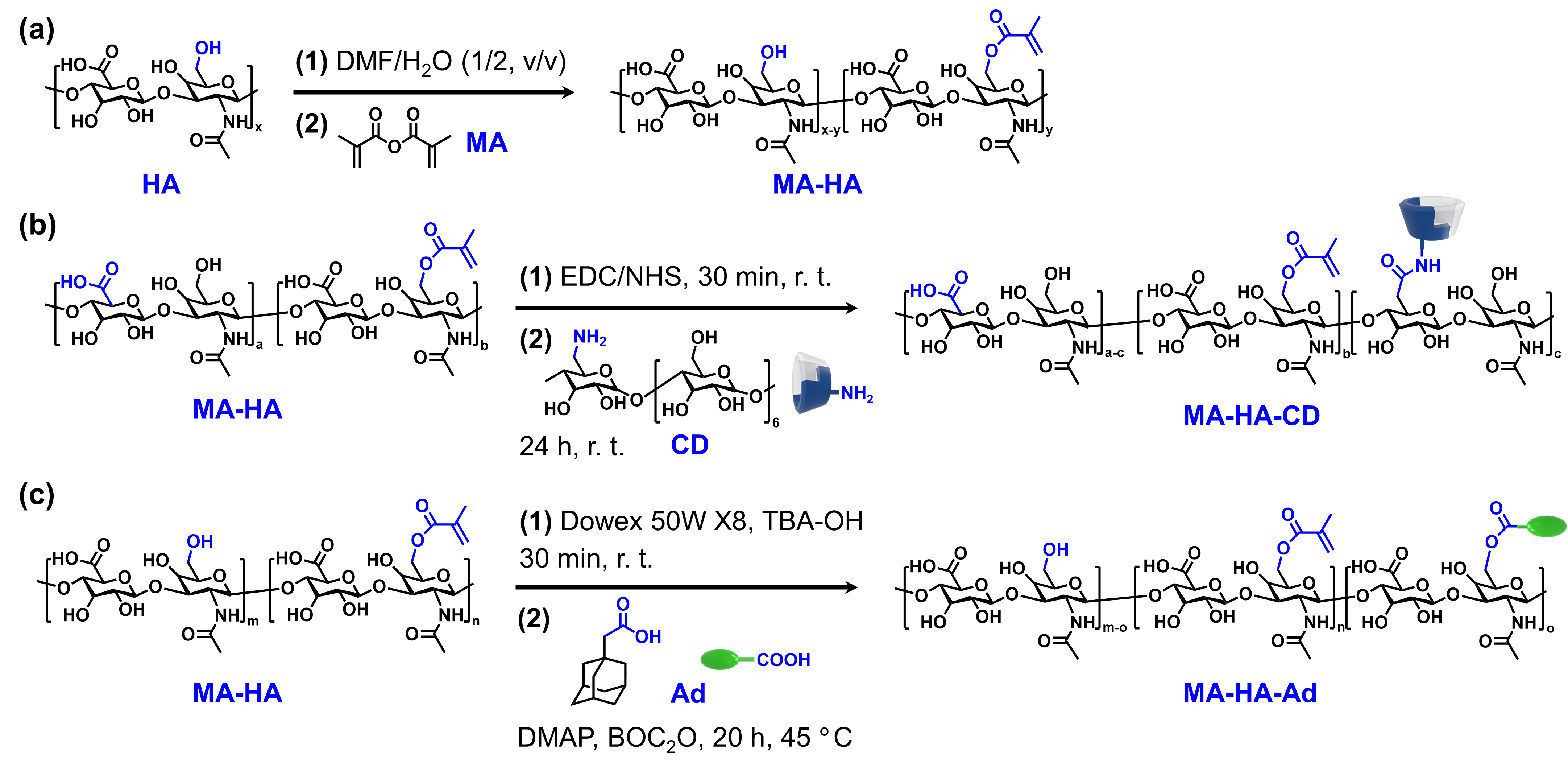


**Scheme S1.** Synthesis routes of (a) MA-HA, (b) MA-HA-CD, and (c) MA-HA-Ad.

*Synthesis of MA-HA* (**Scheme S1a**): 1 g HA (2.7 mmol) was dissolved in 100 mL mixed solvents consisting of DMF and deionized water (v/v=1:2). The reaction system was always kept in an ice-water bath (4 °C). The pH value of the HA solution was adjusted to 8~9 using a NaOH solution (aq, 5 M). Then, 7.4 mL MA (50 mmol) is slowly added dropwise into the HA solution using a dropping funnel, followed by vigorous stirring for 12 h. Afterwards, the mixture was kept to room temperature and stirred vigorously for an additional 12 h to ensure the complete hydrolysis of unreacted methacrylic anhydride. Finally, the reacted mixture was dialyzed for 5 days with frequent water changes each day to remove impurities. Finally, the solution is lyophilized, yielding a white flocculent product.

*Synthesis of MA-HA-CD* (**Scheme S1b**): 500 mg MA-HA was dissolved in 100 mL phosphate buffer (0.2 M, pH = 7.4) (PB). Then, a mixture of EDC (838.5 mg) and NHS (501.5 mg) in 25 mL PB was added, followed by stirring at room temperature for 30 min. 2.5 g CD dissolved in 50 mL PB was added slowly. The mixture was stirred for 24 h at room temperature, followed by dialysis for 5 days and lyophilized. The ^1^H-NMR spectrum of MA-HA-CD in D_2_O is shown in **Figure S1a**. The grafting ratio of CD was calculated to be 7.5% based on the three methyl protons of HA (*H_d_* and *H_d’_* at ~1.8 ppm), the three methacrylate and three methyl protons of MA (*H_a_* at ∼6.1 ppm, *H_b_* at ∼ 5.7 ppm, *H_c_* at ~1.8 ppm), the seven protons on CD ring (*H_f1_* and *H_f1’_* at around 5.0 ppm).

*Synthesis of MA-HA-Ad* (**Scheme S1c**): 3.0 g MA-HA was dissolved in 150 mL deionized water and mixed with 9.0 g ion exchange resin and stirred for 30 min, followed by removing the resin via filtration. The pH of the filtrate was adjusted to neutral using an aqueous solution of tetrabutylammonium hydroxide (TBA-OH). The resulted filtrate was freeze-dried to obtain MA-HA-TBA. Subsequently, MA-HA-TBA (2.50 g, 3.5 mmol), Ad (2.04 g, 10.5 mmol), and DMAP (0.32 g, 2.63 mmol) was mixed in 125 mL anhydrous DMSO and stirred until complete dissolution; then, 0.35 mL BOC_2_O was added. The mixture was reacted at 45 °C for 20 h until adding water to stop the reaction, followed by dialysis in water for 3 d. Some precipitation was observed and filtered. The filtrate was purified by another dialysis for 5 d and lyophilized. The ^1^H-NMR spectrum of MA-HA-Ad in D_2_O is shown in **Figure S1b**. The grafting ratio of Ad was calculated to be 13.6% based on the ratio between 12 protons of Ad (*H_f_* at ~1.5 ppm), and the 10 protons of HA ring (*H_e1-e9_*, *H_e1’_* at 2.9∼4.1 ppm).


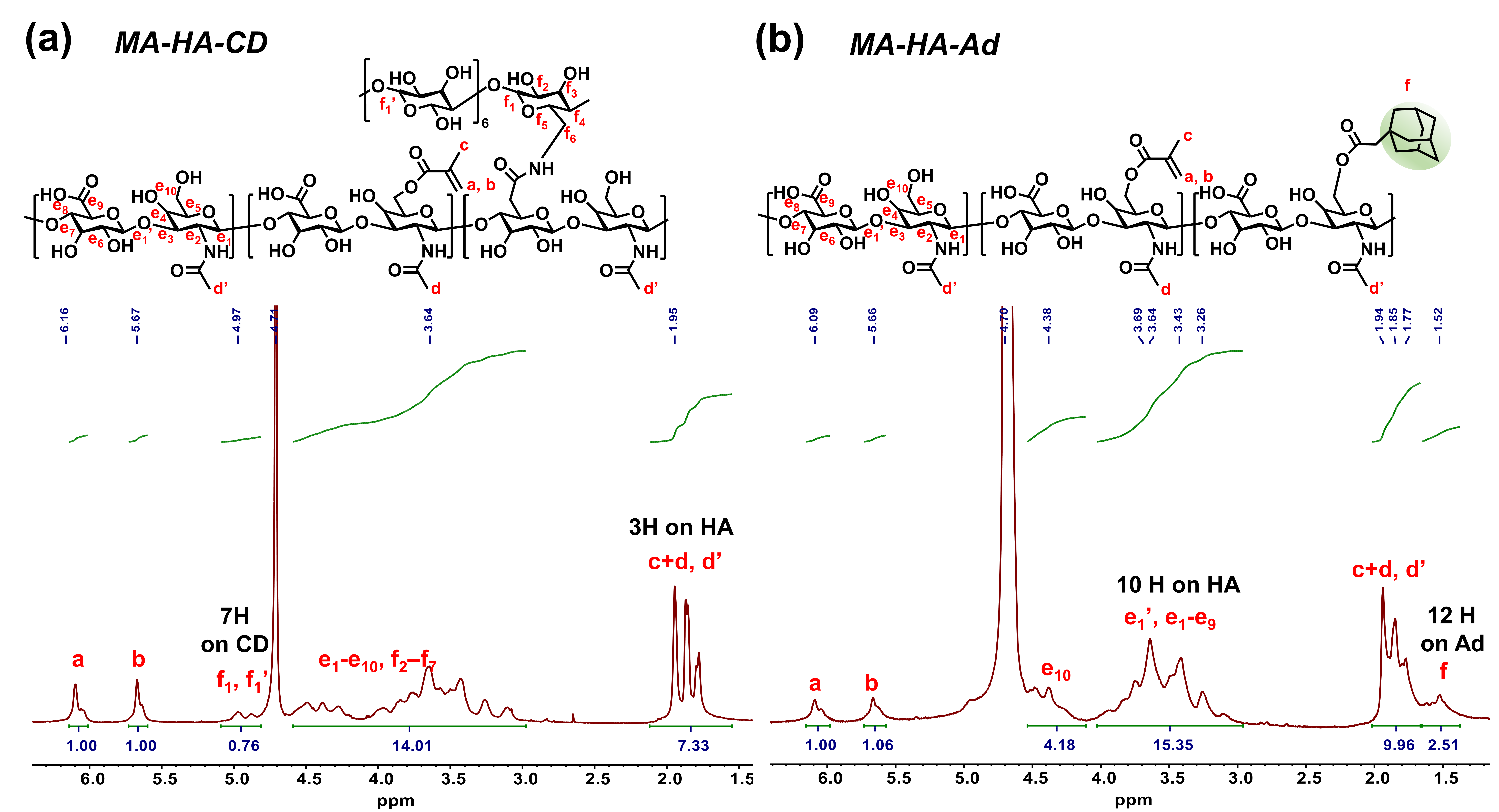


**Figure S1.** ^1^H-NMR spectra of (a) MA-HA-CD and (b) MA-HA-Ad dissolved in D_2_O.

# 3. Fabrication of PDMS Strips


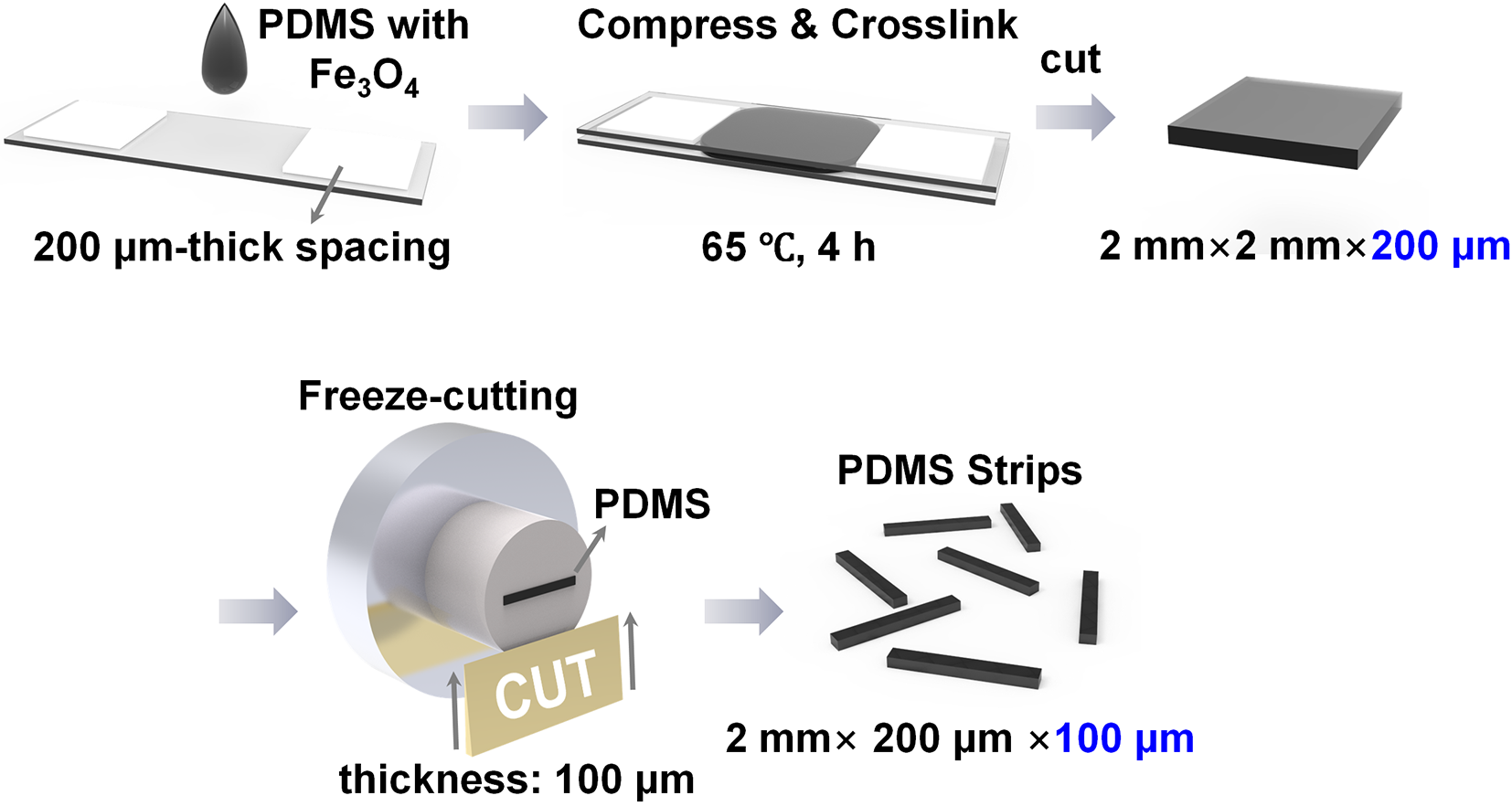


**Figure S2.** Preparation of PDMS strips by sandwiched molding and freeze-cutting.

# 4. Thickness of Polyelectrolyte Multilayers


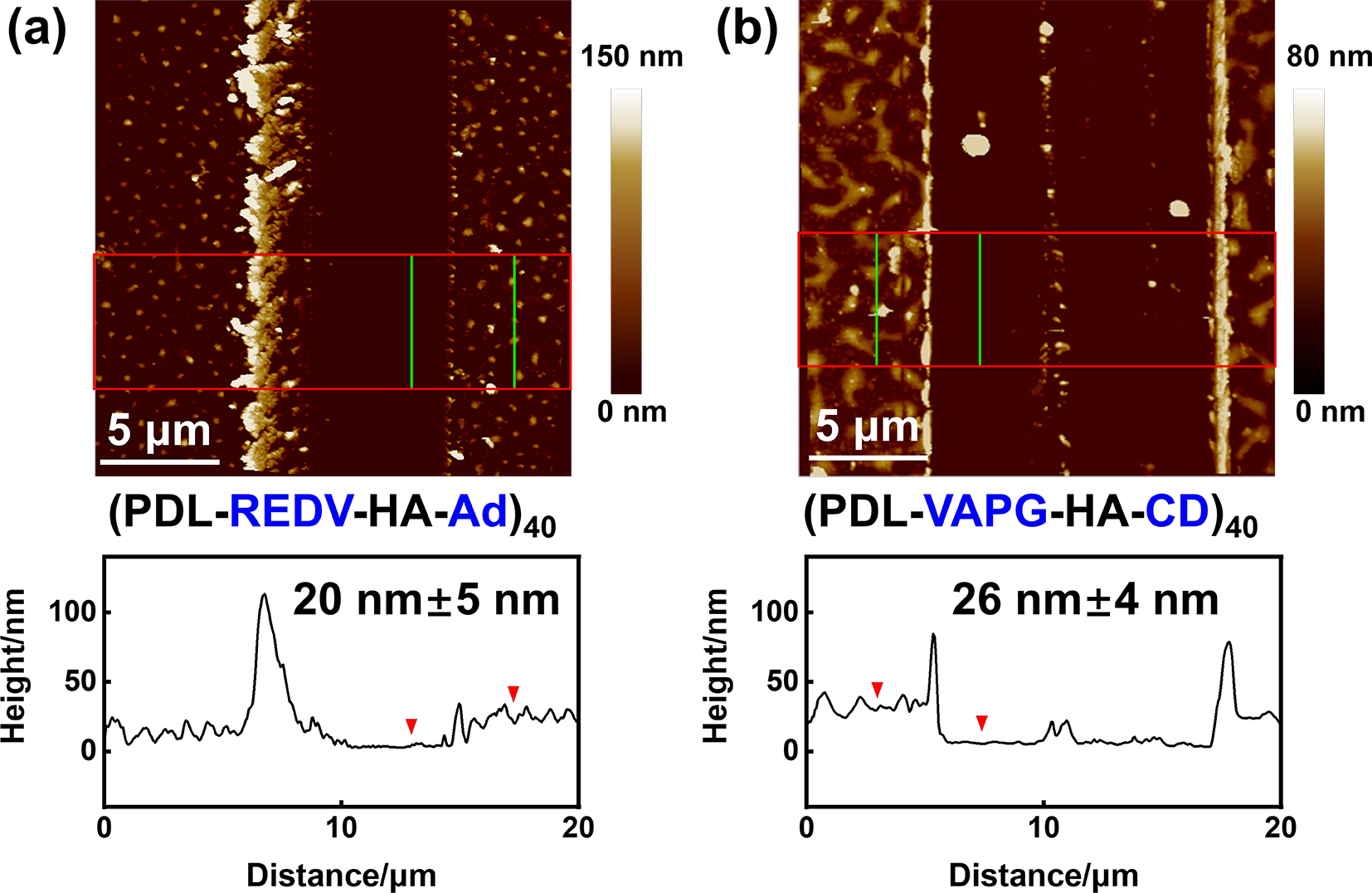


**Figure S3.** AFM images of (a) (PDL/REDV-HA-Ad)_40_ and (b) (PDL/VAPG-HA-CD)_40_ multilayers deposited on quartz substrates and corresponding sectional view to indicate the film thickness.

# 5. Film Stability of (PDL/VAPG-HA-CD)_40_ and (PDL/REDV-HA-Ad)_40_ Multilayers

We detected the stability of (PDL/VAPG-HA-CD)_40_ and (PDL/REDV-HA-Ad)_40_ multilayers deposited on PDMS substrates by immersing them in PBS buffer solutions (pH=7.2~7.4) that contains Na_2_HPO_4_ (8 mM), NaCl (136 mM), and KH_2_PO_4_ (2 mM) for 48 h. The results in **Figure S4** showed that (1) the appearance of the PDMS with coatings remained similar from photos before and after immersion; (2) the hydrophilic surface wettability of the films was maintained according to the water contact angle (WCA) measurements: the PDMS with the (PDL/VAPG-HA-CD)_40_ multilayer has an increased WCA from 29.3 ± 2.7° to 34.0 ± 1.6°; the PDMS with the (PDL/REDV-HA-Ad)_40_ multilayer has an increased WCA from 39.8 ± 1.3° to 45.1 ± 2.3°. By taking the WCA of blank PDMS (109.2 ± 1.2°) as reference, the hydrophobic recovery degree is low, indicating the multilayer coatings remains on PDMS after exposure to PBS for 48 h.


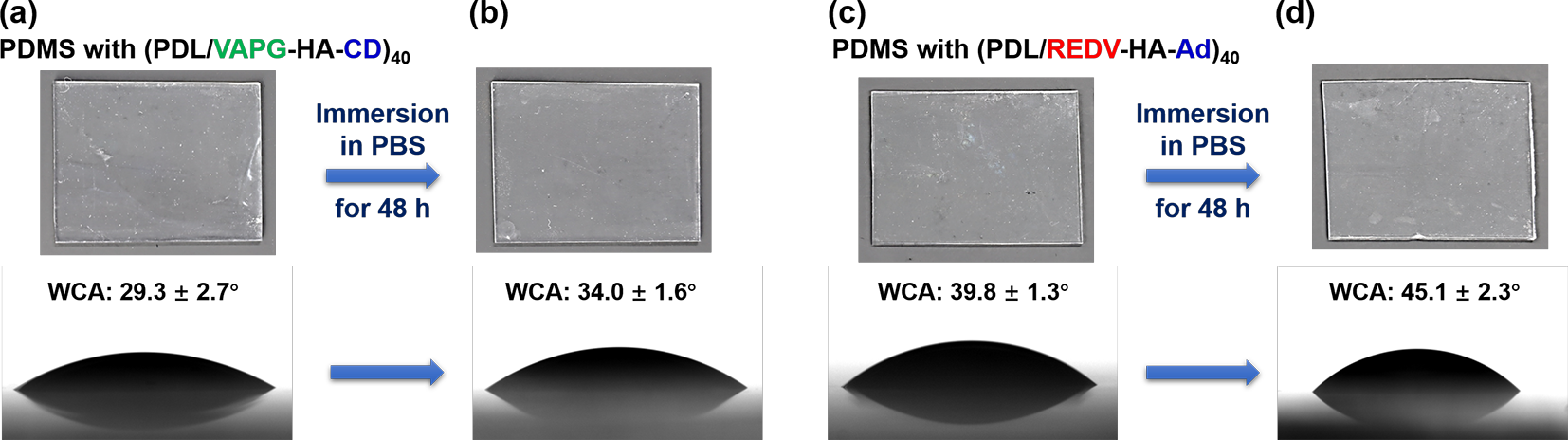


**Figure S4.** Optical appearance of (PDL/VAPG-HA-CD)_40_ and (PDL/REDV-HA-Ad)_40_ multilayers prepared on PDMS substrates (dimension: 50 mm × 20 mm × 1 mm) (a, c) before and (b, d) after immersion in PBS solutions for 48 h.

# 6. Interfacial Interactive Forces between Building Blocks of Varied Surface Chemistry.


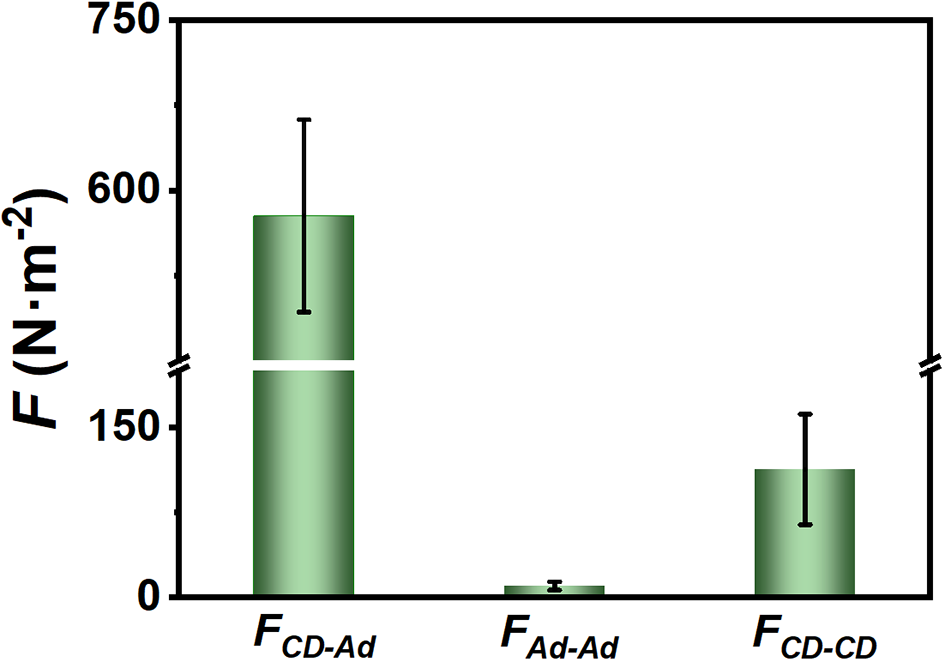


**Figure S5.** Interfacial interactive forces between PDMS modified with (PDL/REDV-HA-Ad)_40_, and (PDL/VAPG-HA-CD)_40_ multilayers, which are noted as Ad and CD for simlicity: *F_CD-Ad_*, means the forces between interactive surfaces while *F_CD-CD_* and *F_Ad-Ad_* represent the forces between surfaces of the same host or guest chemistry.

# 7. Cell Viability of CCS-CD and Cell Adhesion on (PDL/CCS-CD)_40_ Multilayers.


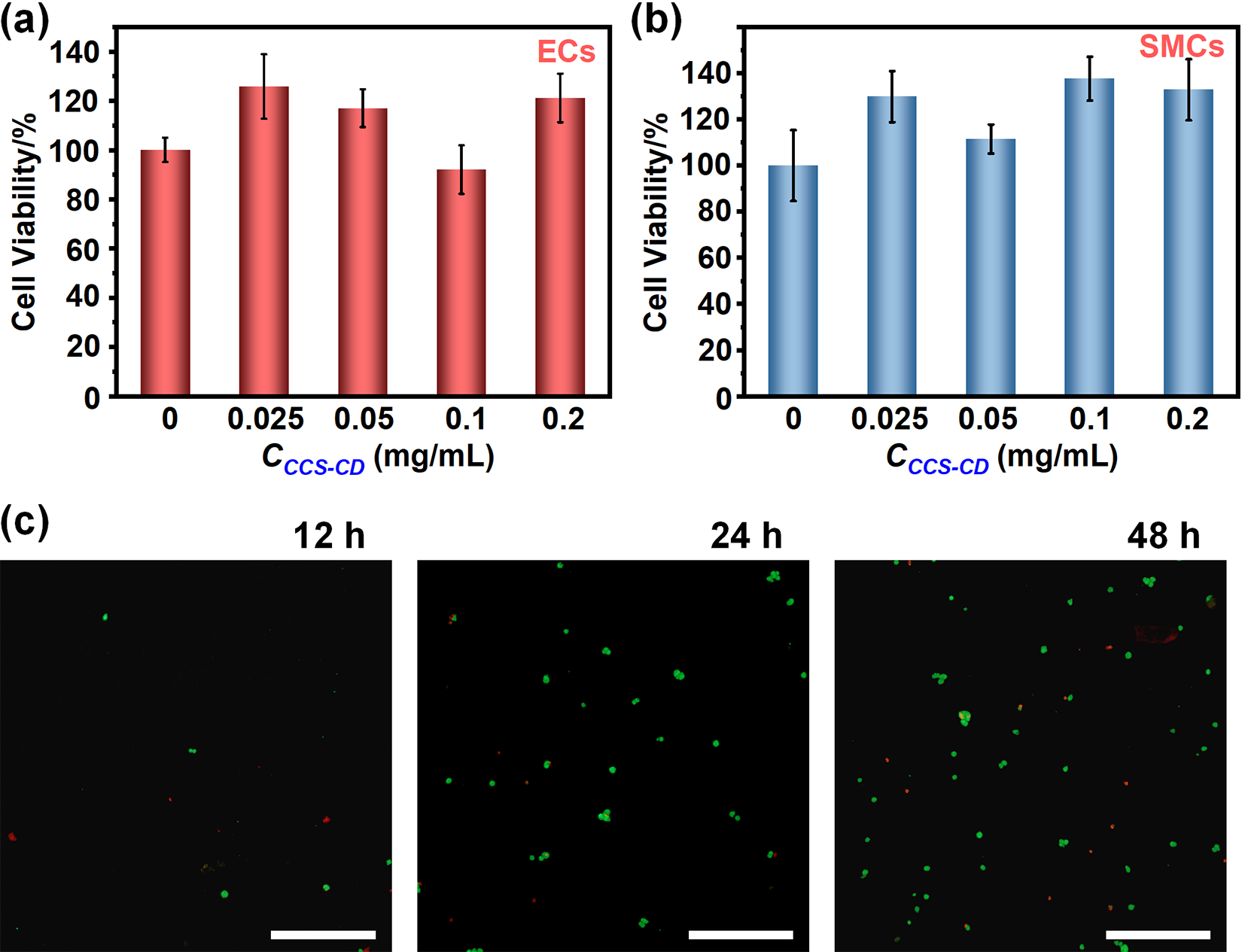


**Figure S6.** MTT results to evaluate the cell viability of (a) ECs and (b) SMCs in CCS-CD. Fluorescence image of ECs cultured on the quartz surfaces modified with (PDL/CCS-CD)_40_ multilayers for 12 h, 24 h, and 48 h. Scale bars: 200 µm.

# 8. Co-Culture of ECs and SMCs on (PDL/REDV-HA-Ad)_40_ Multilayers.


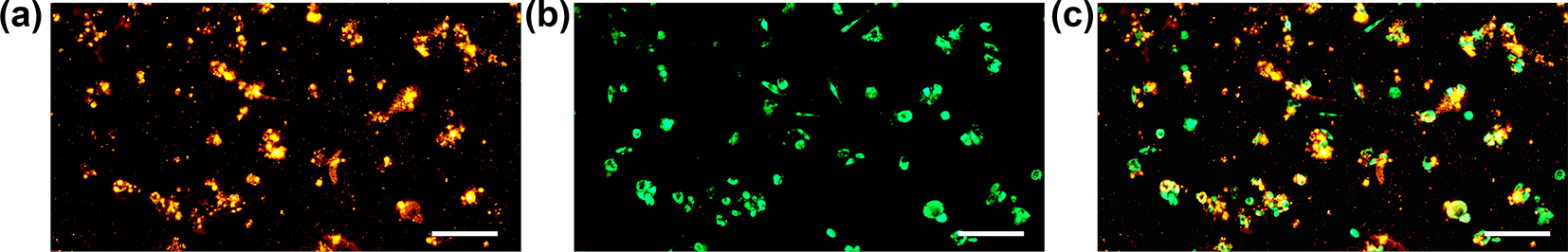


**Figure S7.** Fluorescent images after co-culture of ECs and SMCs on substrates with the surface chemistry of (PDL/REDV-HA-Ad)_40_ multilayers: (a) Dil-labeled ECs, (b) DiO-labeled SMCs, and (c) merged. Scale bars: 200 µm.

# 9. Statistical Analysis of Significant Cell Adhesion Behaviors.


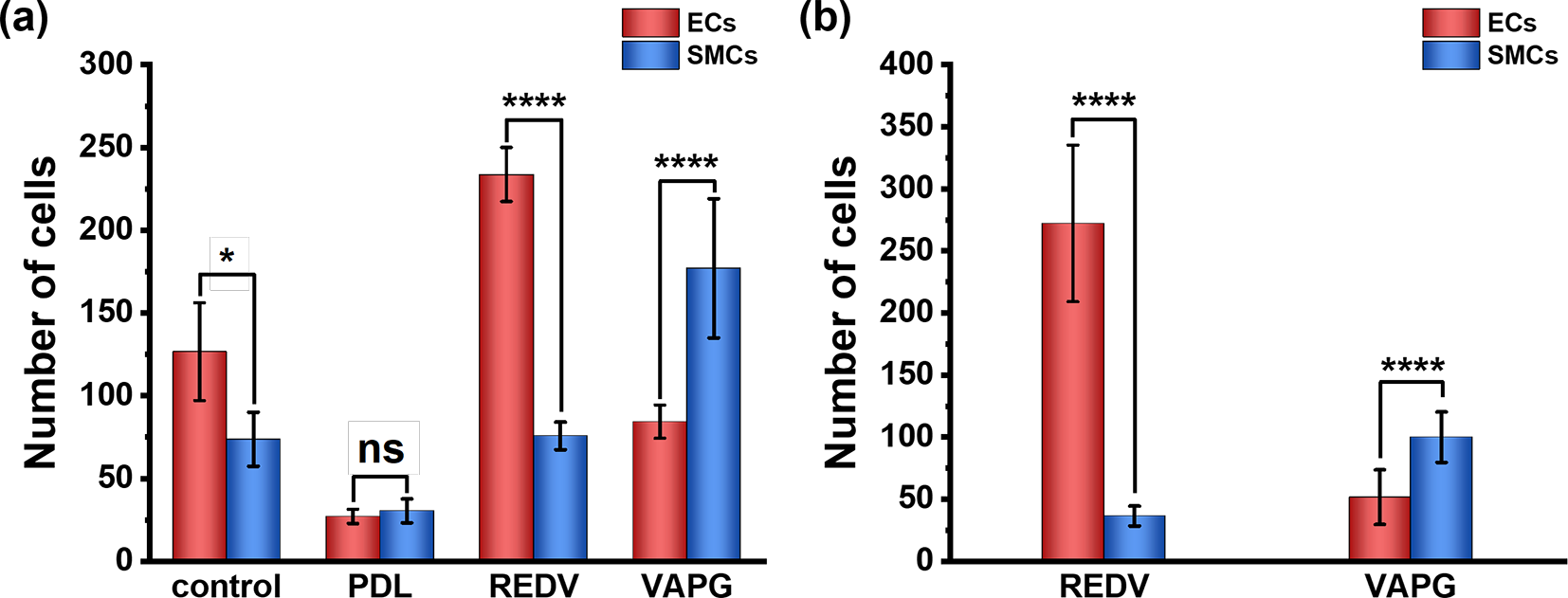


**Figure S8.** Statistical analysis of significant cell adhesion behavior in the results of (a) Figure 5a-c and Figure S7a-c, and (b) Figure 5g following a one-way ANOVA with Tukey’s test (*p < 0.05, **p < 0.01, ***p < 0.001, ****p < 0.0001; ns, not significant).

# 10. Cell Distribution in the Bilayer Structure.


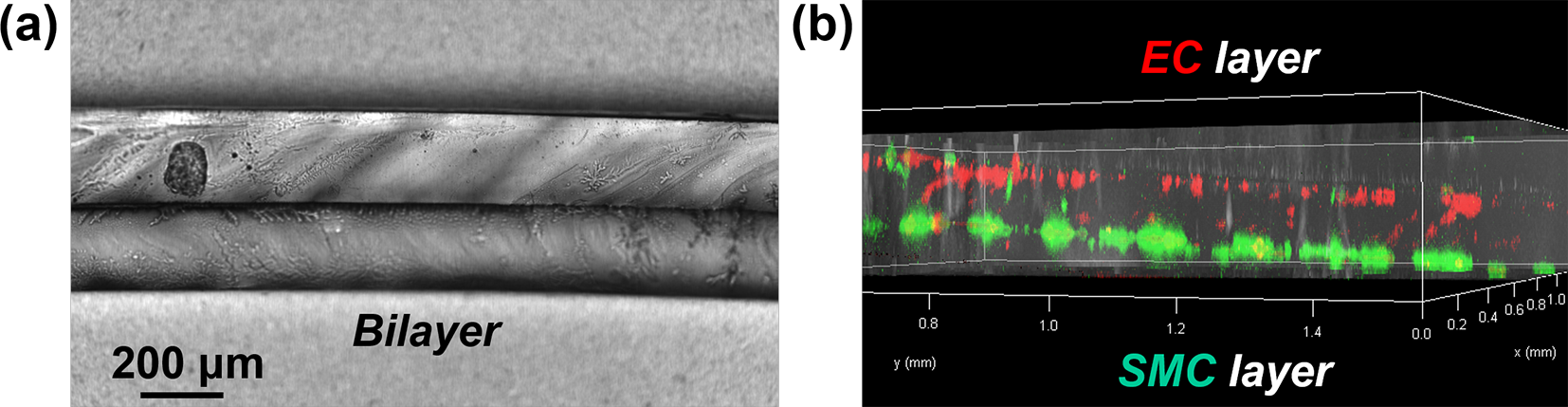


**Figure S9.** (a) Optical microscope and (b) 3D CLSM image of the bilayer PDMS structure in **Figure 5h**.
